# Isolation and characterization of antibody fragments selective for human FTD brain derived TDP-43 variants

**DOI:** 10.1186/s12868-020-00586-0

**Published:** 2020-09-04

**Authors:** Lalitha Venkataraman, Ping He, Galam Khan, Brent T. Harris, Michael R. Sierks

**Affiliations:** 1 School of Life Sciences, Arizona State University, Tempe, AZ USA; 2 Chemical Engineering, School for Engineering, Matter, Transport and Energy, Arizona State University, ECG301-501 Tyler Mall, Tempe, AZ 85281-6106 USA; 3 Departments of Neurology, Georgetown University Medical Center, Washington, DC USA; 4 Departments of Pathology, Georgetown University Medical Center, Washington, DC USA

**Keywords:** Frontotemporal dementia, TDP-43 variants, scFv, Biomarker, Brain tissue, Sera

## Abstract

**Background:**

Frontotemporal dementia (FTD) is the second leading cause of early onset dementia following Alzheimer’s disease. It involves atrophy of the frontal and temporal regions of the brain affecting language, memory, and behavior. Transactive response DNA-binding protein 43 (TDP-43) pathology is found in most FTD and ALS cases. It plays a role in transcription, translation and serves as a shuttle between the nucleus and cytoplasm. Prior to its aggregation, TDP-43 exists as polyubiquitinated, hyperphosphorylated C-terminal fragments that correlate well with FTD disease progression. Because of the importance of TDP-43 in these diseases, reagents that can selectively recognize specific toxic TDP variants associated with onset and progression of FTD can be effective diagnostic and therapeutic tools.

**Results:**

We utilized a novel atomic force microscopy (AFM) based biopanning protocol to isolate single chain variable fragments (scFvs) from a phage display library that selectively bind TDP variants present in human FTD but not cognitively normal age matched brain tissue. We then used the scFvs (FTD-TDP1 through 5) to probe post-mortem brain tissue and sera samples for the presence of FTD related TDP variants. The scFvs readily selected the FTD tissue and sera samples over age matched controls. The scFvs were used in immunohistochemical analysis of FTD and control brain slices where the reagents showed strong staining with TDP in FTD brain tissue slice. FTD-TDP1, FTD-TDP2, FTD-TDP4 and FTD-TDP5 all protected neuronal cells against FTD TDP induced toxicity suggesting potential therapeutic value.

**Conclusions:**

These results show existence of different disease specific TDP variants in FTD individuals. We have identified a panel of scFvs capable of recognizing these disease specific TDP variants in postmortem FTD tissue and sera samples over age matched controls and can thus serve as a biomarker tool.

## Background

Frontotemporal dementia (FTD) is the second leading cause of early onset dementia following Alzheimer’s disease [1]. FTD is diverse and involves atrophy of the frontal and temporal regions of the brain affecting language, memory and personality [2]. Based on prominent pathological protein inclusions of TDP-43, tau or Fused in Sarcoma (FUS), FTD is classified as either FTD-TDP, FTD-tau or FTD-FUS [3]. Studies have shown that these subtypes have overlapping molecular pathology, making diagnosis difficult [4–6], despite progress in imaging techniques and CSF biomarkers.

There are currently several imaging techniques like diffusion tensor imaging, fMRI (voxel based changes) and PET scan that have shown promise in FTD diagnosis [7–9]. These techniques have been demonstrated on a small scale and are focused on measuring anatomical similarities within FTD and assessing differences between FTD and other dementias. Relying only on imaging for diagnosis has limited potential since FTD falls under a spectrum with a wide range of anatomical representations.

Apart from imaging, current CSF based biomarkers for FTD focus on measuring p-tau, tau and Aβ42 which is similar in certain FTD subtypes compared to AD [10]. While a fraction of FTD cases demonstrate AD pathology [11–14], over 50% of Alzheimer’s cases present with TDP pathology [15–18] rendering AD based biomarkers (p-tau and Aβ) unreliable. Therefore, there is a need for biomarkers that can differentiate FTD from other diseases.

Although there is a familial component to FTD with mutations identified in MAPT, chromosome 9 open reading frame 72 (C9orf72) and GRN, extensive TDP-43 pathology has been observed in both familial and sporadic cases of FTD [19–21]. TDP-43 is a TAR DNA binding nuclear protein, 414 amino acids in length coded by the TARDBP gene. TDP-43 is a common molecular pathology in the FTD-ALS spectrum and is observed in more than 50% of FTD cases [22]. It plays a key role in transcription and translation processes and is involved in alternate splicing, mRNA transport and serves as a shuttle between the nucleus and cytoplasm [23]. In FTD, TDP-43 is translocated to the cytoplasm [24] and the location and type of aggregates present [25] differ in clinical subtypes of FTD [5]. Elevated levels of TDP 43 are found in circulating CSF of FTD and ALS patients [24, 26]. Although the pathogenic mechanisms is not known, several studies indicate that TDP-43 can spread in a prion like fashion from neuron to neuron through the axonal pathway [27–31]. TDP-43 is also implicated in ALS, where different strains of TDP-43 have been shown to spread at different rates in in vitro models, indicating presence of multiple toxic TDP variants [27, 32]. Different TDP-43 conformations with different levels of toxicity resulting in different pathologies (TDP type A-D) and disease phenotype have been identified [33]. These TDP-43 variants exist due to post-translational modifications such as hyperphosphorylation, polyubiquitination and truncation leading to C-terminal fragments that are toxic [24, 34–38]. Currently, there is a lack of accurate blood-based biomarkers for FTD irrespective of familial or sporadic origin. We hypothesize that FTD specific TDP-43 variants can be used as unique biomarkers in early antemortem diagnosis distinguishing FTD from other neurodegenerative diseases. We have identified a unique panel of scFvs capable of recognizing TDP variants that are present in human FTD patients but not in age-matched cognitively normal controls.

## Results

### Phage and scFv purification

**Fig. 1:**
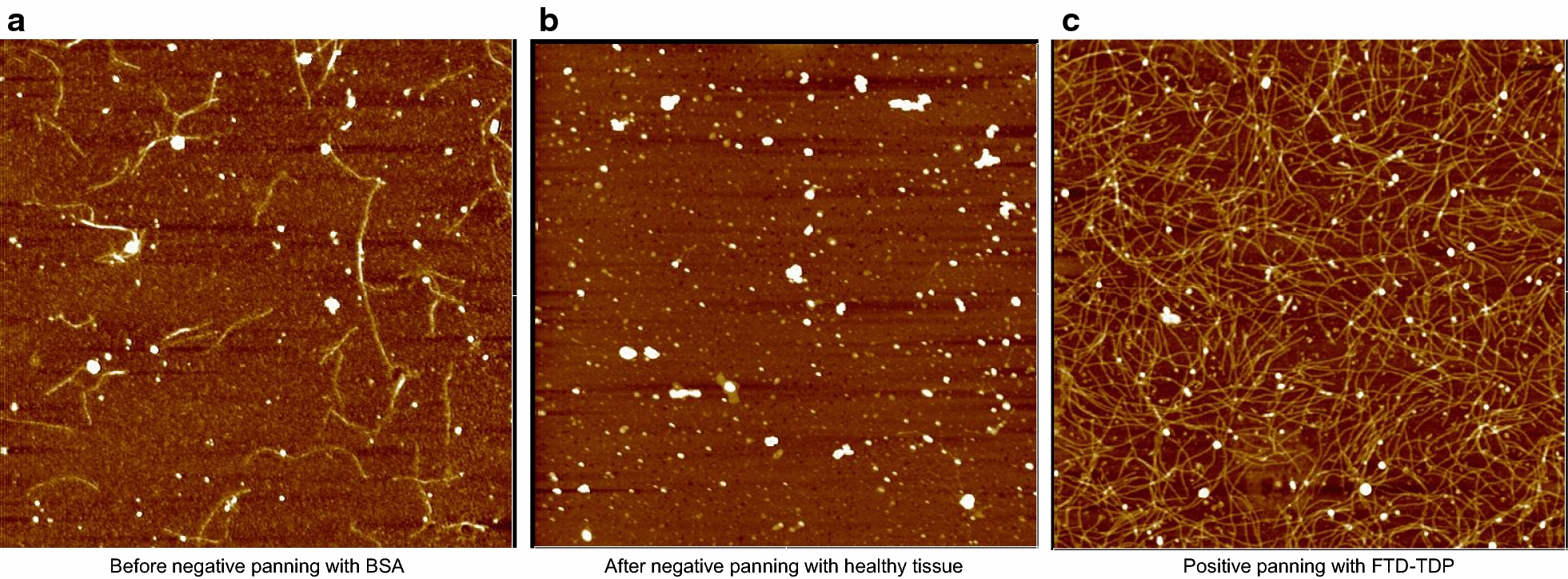
AFM panning images. Atomic Force Microscopy images of (**a**) phage binding to BSA prior to subtractive panning to get rid of non-specific binders, (**b**) no phage binding is observed after multiple rounds of subtractive panning with healthy control tissue, (**c**) phage binding with FTD-TDP IP after positive selection

**Fig. 2:**
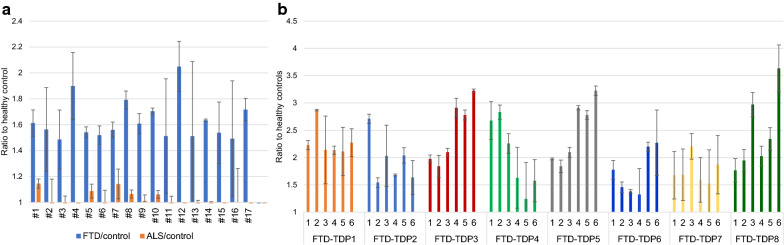
Screening of anti-TDP phage with pooled and individual FTD and control brain tissue homogenates. Phage obtained after positive selection against human FTD brain derived TDP variants were screened with (**a**) pooled human FTD brain tissue samples (n = 3), pooled ALS brain tissue samples (n = 3) and healthy controls (n = 2), (**b**) Selected 8 phages were further screened with individual FTD brain tissue homogenates (n = 6) and healthy controls. Error bars based on SEM

After serial rounds of subtractive panning were performed to remove phage that bound off target antigens including BSA, homogenate from healthy human brain tissue and TDP-43 immunoprecipitated from pooled ALS brain tissue homogenates, a single round of positive selection was performed using immunoprecipitated TDP-43 from pooled FTD brain tissue (Fig. 1). Eighty phage clones were recovered from the positive panning step and were verified for sequence integrity by DNA sequencing. The reactivity of 17 phage clones were further tested to verify that they bound human FTD brain tissue, but not ALS or healthy brain tissue homogenates using pooled tissue homogenates. All 17 phage preparations showed high reactivity to the FTD sample with very little or no reactivity to the ALS sample (Fig. 2a).

Based on the initial ELISA screening, we selected eight phage clones with the highest reactivity with pooled FTD but no reactivity with pooled ALS and pooled healthy control tissue homogenates for further testing with individual FTD (n = 6) and age-matched cognitively normal (n = 2) brain homogenates (Fig. 2b). The 8 phage samples reacted with each of the FTD brain tissue homogenates, however each phage had a different binding pattern among the FTD patients suggesting that they bind different TDP variants.

**Fig. 3:**
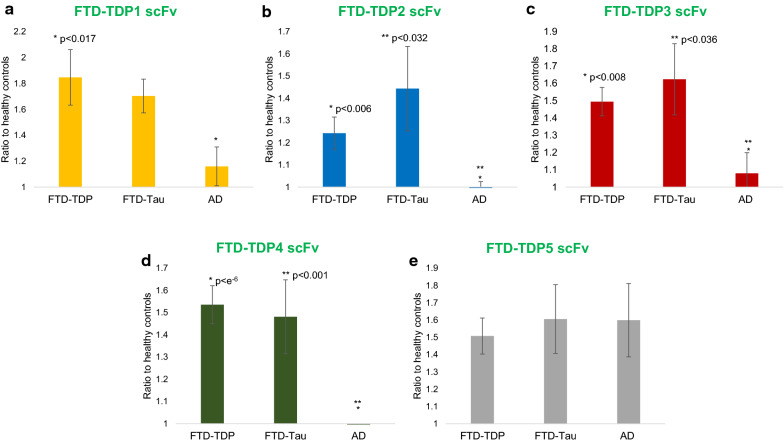
Anti-TDP scFvs characterization with FTD and control sera. Reactivity of anti-TDP scFvs with FTD and AD sera were assessed using sandwich ELISA. 4 of the 5 anti-TDP scFvs selectively bind to both FTD-TDP (n = 12) and FTD-tau (n = 12) sera and has relatively little to no binding to AD sera (n = 11). FTDP-TDP5 was the only scFv that had reactivity with FTD-TDP, FTD-Tau and AD sera over cognitively normal healthy controls. Error bars based on SEM

The five phage clones that showed the strongest reactivity toward the individual FTD tissue samples and lowest reactivity towards the control samples were expressed as scFvs and used to determine if the TDP variants could also be detected in sera samples. The five scFvs (FTD-TDP1, FTD-TDP2, FTD-TDP3, FTD-TDP4 and FTD-TDP5) were used to assay sera samples from FTD-TDP (n = 12), FTD-tau (n = 12), AD sera (n = 11) and controls (n = 10) (Fig. 3). Four of the scFvs (FTD-TDP1, FTD-TDP2, FTD-TDP3 and FTD-TDP4) have significantly higher reactivity to FTD-TDP and FTD-tau sera samples compared to AD sera samples, while the fifth scFv (FTD-TDP5) had high reactivity with all the FTD and AD samples. None of the scFvs studied here discriminated between FTD-TDP and FTD-tau sera samples, though four of them did discriminate between FTD and AD samples suggesting that some TDP variants are unique to FTD, while others are involved in both FTD and AD. The sensitivity and specificity of each of the five anti-TDP scFvs for FTD-TDP and FTD-tau are shown (Table 1). All the scFvs have area under curve (AUC) > 0.84 implying high sensitivity and specificity of the scFvs in selecting FTD sera over healthy controls.

**Table 1:** Sensitivity and specificity of five anti-TDP FTD scFvs based on reactivity with FTD-TDP (n = 12), FTD-tau (n = 12) and control sera (n = 8)

scFv	FTD-TDP			FTD-Tau			Total		
	Sensitivity (%)	Specificity (%)	AUC	Sensitivity (%)	Specificity (%)	AUC	Sensitivity (%)	Specificity (%)	AUC
FTD-TDP1	91.66	100	0.99	75	75	0.85	75	87.5	0.92
FTD-TDP2	75	100	0.87	75	100	0.82	75	100	0.84
FTD-TDP3	100	87.5	0.99	91.66	87.5	0.96	95.83	100	0.99
FTD-TDP4	100	83.3	0.97	75	75	0.79	75	87.5	0.88
FTD-TDP5	100	100	1	100	100	1	100	100	1

Sensitivity and specificity were calculated for FTD-TDP, FTD-tau and both FTD subtypes–AUC values greater than 0.95 are highlighted

### Western blot analysis

**Fig. 4:**
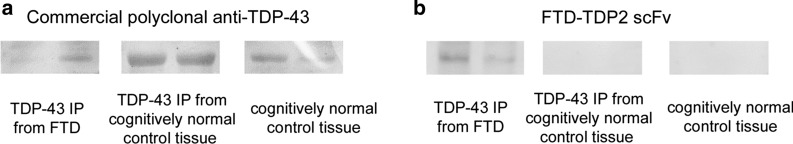
Western Blot Analysis. Reactivity against healthy control tissue and TDP-43 immunoprecipitated from healthy controls and FTD was assessed under non-reducing and non-denaturing conditions with (**a**) Commercial TDP antibody identifying TDP variants in FTD and healthy controls, and (**b**) FTD-TDP2 scFv which recognizes disease variant of TDP (~ 70 kDa) present in FTD and not healthy controls

We assume that the FTD selective scFvs bind conformational epitopes of TDP-43 that are involved in FTD since the scFvs did not bind TDP variants present in healthy age-matched control tissue. To verify that the scFvs were binding a conformational epitope, we analyzed PAGE gels under denaturing (Additional file 1: Fig. S1) and native conditions by probing with a commercial anti-TDP antibody and the FTD-TDP2 scFv (Fig. 4, Additional file 1: Fig. S2). Under native conditions, FTD-TDP2 scFv recognizes a disease variant of TDP-43 present in FTD (~ 70 kDa) but not in healthy control tissue or TDP-43 immunoprecipitated from healthy control tissue.

### Competition ELISA

**Fig. 5:**
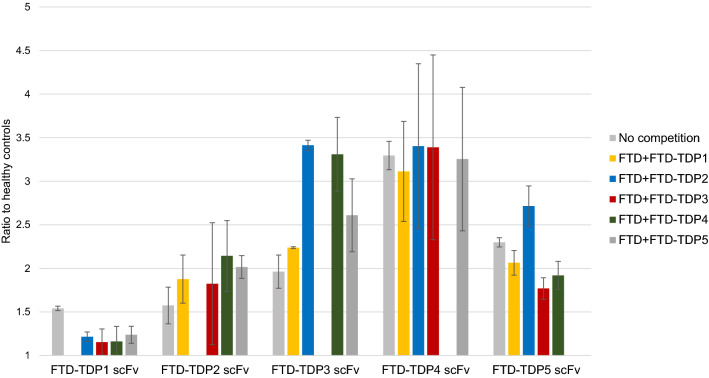
Competition ELISA of anti-TDP scFvs. X-axis represents each scFv and Y-Axis resents ratio to age matched controls. Each scFv was tested with FTD sera (1 FTD-TDP +1 FTD-tau) (no competition) or FTD sera pre-incubated with each of the other four scFvs (competition). One-way ANOVA analysis indicate no significant difference between the no competition and competing scFvs. Error bars based on SEM

To determine if the five different scFvs against the FTD related TDP variants were binding different epitopes, we performed a competition ELISA where each scFv was tested with FTD sera (no competition) or FTD sera preincubated with one of the other 4 scFvs (competition) (Fig. 5). If any two scFvs recognize the same epitope, we expect a significant reduction in ELISA signal. One-way ANOVA analysis indicated there was not any difference between the control samples and those with added scFv indicating that the scFvs bind unique epitopes.

### Immunohistochemistry

**Fig. 6:**
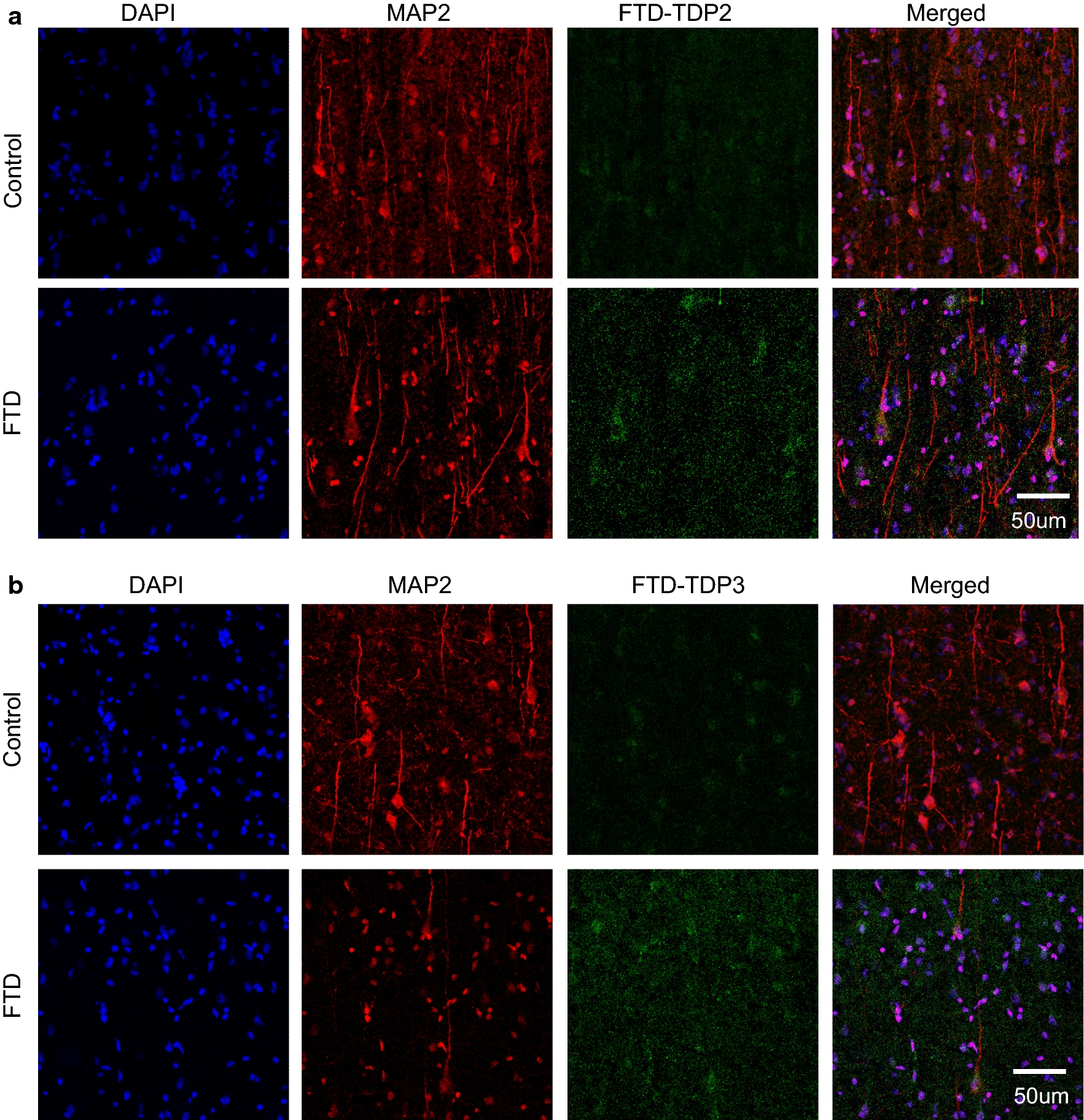
Immunohistochemistry with anti-TDP scFvs. Tissue sections were incubated with (**a**) FTD-TDP2, or (**b**) FTD-TDP3 (1:100) on a shaking stage overnight at 4 °C. Primary antibodies against c-myc region of scFv (Sigma, 1:1000, rabbit) and MAP2 (Covance, 1:400, mouse) were applied followed by goat anti-rabbit IgG (green) and goat anti-mouse IgG (red) with fluorescence. The sections were observed and imaged with Leica SP5. Scale bar represent 50 µm

Two anti-TDP scFvs were further studied using IHC analysis of human postmortem FTD and control brain tissue sections. The FTD-TDP2 and FTD-TDP3 scFvs were utilized for the IHC analyses since they had high expression levels and high reactivity with FTD over cognitively normal controls in tissue and sera analyses. Fluorescence tagged secondary antibodies were used to visualize the microtubule associated protein (MAP2) (red) and the bound scFvs (green) (Fig. 6). Although MAP2 staining is present in both FTD and control cases, there is no anti-TDP scFv staining with the control case. In the FTD case, there is extensive anti-TDP scFv staining indicating that the FTD-TDP scFvs recognize disease specific TDP variants. Both the anti-TDP scFvs have similar staining surrounding the nucleus (blue) in FTD tissue indicating presence of intraneuronal TDP variants in the cytoplasm.

### Toxicity assay

**Fig. 7:**
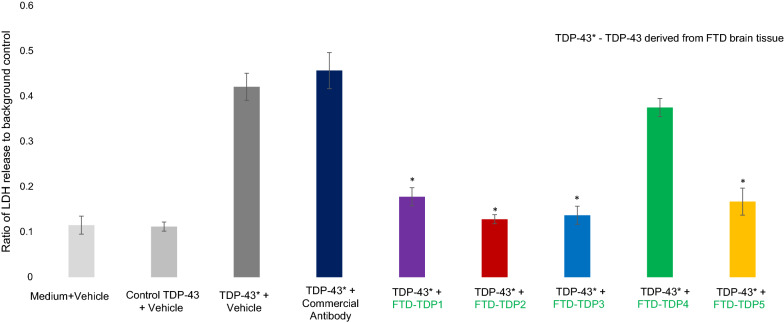
Therapeutic potential of anti-TDP scFvs. SH-SY5Y neuroblastoma cell line was treated with TDP-IP derived from human FTD and control brain tissue. The cells were further treated with a commercial anti-TDP antibody (ab190963, Abcam, 1 µg/mL) or anti TDP scFvs (FTD-TDP1, FTD-TDP2, FTD-TDP4 and FTD-TDP5) for 12 h. The cell damage and toxicity were tested by measuring lactate dehydrogenase (LDH). Neither the commercial TDP antibody or FTD-TDP4 significantly blocked toxicity of the FTD brain derived TDP IP at the concentration studied, while FTD-TDP1, FTD-TDP2, FTD-TDP3 and FTD-TDP5 all significantly inhibited the TDP induced toxicity. Error bars based on SEM

When incubated with neuronal cells, the TDP-43 sample immunoprecipitated from human post-mortem FTD brain tissue induced significantly increased toxicity toward cultured SH-SY5Y cells compared to TDP-43 immunoprecipitated from cognitively normal human brain tissue (Fig. 7). Five anti-TDP scFvs with high selectivity for FTD sera over the controls (FTD-TDP1, FTD-TDP2, FTD-TDP4 and FTD-TDP5) and a commercial antibody against TDP-43 were separately co-incubated with the cells at a concentration of 1 µg/mL to block TDP variants from interacting with the cells. Three of the scFvs, FTD-TDP1, FTD-TDP2 and FTD-TDP5 significantly reduced toxicity of the TPD-43 while the commercial anti-TDP-43 antibody did not reduce toxicity and the FTD-TDP4 scFv only slightly decreased toxicity at the concentration studied. These results indicate that FTD related TDP variants are toxic to neuronal cells, and that selectively targeting the TDP variants may be an effective therapeutic for treating FTD and potentially other related neurodegenerative disease.

## Discussion

TDP-43 pathology is commonly observed in a vast number of FTD cases and TDP-43 variants are observed in CSF and sera making it an ideal candidate for antemortem FTD diagnosis [26, 39–42]. We generated a panel of scFvs that selectively bind FTD specific TDP-43 variants using an AFM-based biopanning protocol [43]. Five scFvs that had high reactivity with individual FTD brain tissue over control tissue (FTD-TDP1, FTD-TDP2, FTD-TDP4 and FTD-TDP5) were further analyzed using sera samples from FTD-TDP, FTD-tau, AD, and control cases (Figs. 1, 2, 3). Four of the five scFvs tested showed high reactivity with FTD sera but not AD or cognitively normal controls, while one scFv showed high reactivity with the FTD and AD sera cases (Fig. 3). Even though around 50% of AD cases have prominent TDP pathology [15, 16], it is apparent that TDP pathology in FTD cases has some distinct differences from that in AD cases. Although FTD sera has been classified as FTD-TDP and FTD-tau based on postmortem pathology reports, studies have shown that there is an overlap of tau and TDP-43 pathology in FTD cases [44]. Here we also observed that TDP pathology between the FTD-TDP and FTD-tau cases are quite similar (Figs. 2, 3).

TDP-43 undergoes several post-translational modifications and occurs as C-terminal fragments of varying lengths [37, 45]. Previous studies have indicated that a 70 kDa species is present in FTD brain tissue studies [38]. Here, we show that FTD-TDP2 scFv recognizes a conformation specific 70 kDa species present in FTD and not in cognitively normal healthy control tissue samples (Fig. 4) and that this variant is localized in the cytoplasm of neurons in FTD brain tissue but not healthy controls (Fig. 6). Other neurodegenerative diseases like motor neuron disease, AD, dementia with Lewy bodies and Huntington’s disease also exhibit TDP pathology [46–48]. We also showed that the TDP variants present in FTD brain are toxic to neuronal cells (Fig. 7), and that selectively targeting the toxic variants may be an effective therapeutic option for treating FTD and other TDP related diseases. Investigating overlap in TDP pathology in these different diseases and further investigating therapeutic potential of these reagents will be the focus of future studies.

## Conclusion

We have generated a panel of scFvs that selectively bind TDP-43 protein variants present in postmortem FTD brain tissue and sera samples but not age matched, healthy controls. These results indicate the diagnostic potential of these scFvs in distinguishing FTD from healthy controls and other TDP-43 pathologies.

## Methods

### Human specimens

Human brain tissue homogenates from motor cortex of FTD (n = 3), ALS (n = 3) and healthy controls (n = 2) and immunoprecipitated TDP-43 from these pathologically validated cases were provided from Georgetown Brain Bank (Georgetown University Medical Center). These samples were used in the initial AFM based screening. Human postmortem brain tissue sections from the superior frontal cortex and sera samples from FTD and control were provided by Dr. Thomas Beach, director of the Brain and Body Donation Program at Banner Sun Health Research Institute (BBDP) [49, 50]. The brain sections were used for immunohistochemistry studies and sera samples (FTD-TDP (n = 12), FTD-Tau (n = 12), AD (n = 11)) used in ELISA characterization studies.

### Panning using immunoprecipitated TDP-43

Frozen brain tissue samples were briefly homogenized as described previously [51]. Briefly tissue was sonicated in cold lysis buffer: 25 mM HEPES NaOH (pH 7.9), 150 mM NaCl, 1.5 mM MgCl2, 0.2 mM EDTA, 0.5% Triton-X-100, 1 mM dithiothreitol, protease inhibitor cocktail. The homogenized sample was centrifuged, and the supernatant was frozen in − 80 °C.

TDP-43 protein was immunoprecipitated from brain tissue homogenates which were pooled (3 FTD samples and 2 healthy controls) using a commercial polyclonal antibody against TDP-43 protein (ProteinTech Inc, Chicago, IL; Catalog # 10782-2-AP) as validated in [52]. The immunoprecipitated samples were probed with 1:1000 dilution of commercially available anti-TDP antibody (ProteinTech Inc, Chicago, IL; Catalog # 10782-2-AP) to verify the presence of TDP-43.

A combination of commercially available phage display libraries–Sheets, Tomlinson I and Tomlinson J with a variability of 10^8^ and concentration of 10^12^ pfu/m were used for the panning [53, 54]. We utilized an AFM based selection process that uses exhaustive subtractive panning steps to remove non-specific phage binding clones as well as clones binding to off-target antigens including antibody fragments that bound to TDP-43 forms from healthy individuals and from ALS patients as described previously [43]. Atomic force microscopy (AFM) imaging was performed after every subtractive panning step to ensure removal of all antibody fragments binding these off-target antigens. Phage that did not bind to any of the off-target antigens was used for the final positive selection round performed against TDP immunoprecipitated from pooled FTD brain tissue samples. For this positive panning step, the TDP IP preparation was deposited on mica since only nanogram quantities of the antigen are needed and the process can be monitored via AFM imaging. Phage were eluted using trypsin and TEA and grown on LB–Amp plates overnight at 37 °C.

### Phage and scFv purification

Phage obtained after the positive selection were sequenced to ensure that they encoded complete scFv sequences. After sequence validation, phage were amplified as described [54]. Phage titers were performed to verify the concentration of phage (~ 10^9^ pfu/mL). Soluble scFv were also prepared by transforming the plasmids from each phage into *E. Coli* strain HB2151. An overnight culture was used for growing scFv in 2xYT media at 37 °C for 3–4 h. The scFvs were grown and purified using a protein A Sepharose affinity column (GE Healthcare) as described [43, 55]. Molecular size of the scFvs were checked in both the supernatant and lysate fraction via western blot with 1:2000 dilution of anti-c-myc 9e10 primary antibody (SantaCruz; Catalog # sc-40) followed by 1:2000 dilution of secondary antibody goat anti-mouse HRP (SantaCruz; Catalog # sc-2005). The DNA sequences of the scFvs were also validated using MAFFT, a multiple sequence alignment software.

### TDP phage biotinylation

An aliquot of the remaining phage pool that was recovered after exhaustive subtractive panning with BSA, and aggregated α-synuclein and TDP-43 immunoprecipitated from healthy control tissue was used to select a detection phage for sandwich ELISA [43]. A phage expressing an scFv that binds to all forms of TDP-43 contained in both FTD and ALS samples was selected to increase signal to noise ratio in ELISA. This phage was biotinylated using the EZ-Link Pentylamine-Biotinylation kit (Thermo Scientific, USA) as described [56]. The detection phage binds TDP variants present in both FTD and ALS samples and does not compete for the same binding sites as the capture scFv in sandwich ELISA.

### FTD tissue and sera analysis

Brain tissue homogenates from FTD (n = 3), ALS (n = 3) and healthy individuals (n = 2) were pooled together and used for the initial screening assay as described previously [56]. The pooled brain tissue homogenate was used to coat the plates and tested for reactivity with each of the phages. This assay was used to evaluate binding specificity of all the phage clones for FTD over ALS and cognitively normal control samples.

Soluble antibody fragments (scFv) (FTD-TDP1, FTD-TDP2, FTD-TDP3, FTD-TDP4 and FTD-TDP-5) were produced for each of the phages that had a high signal with the FTD brain tissue homogenates in the indirect ELISA. The scFvs were used as the capture antibody in a sandwich ELISA to test reactivity with sera samples (12 FTD-TDP, 12 FTD-tau and 10 healthy controls) as described [43, 56, 57]. The bound species was detected using biotinylated TDP phage to amplify the signal to noise ratio. Signal ratios were determined by comparing the signal obtained for each scFv with the FTD sera to healthy controls and plotted as described [43].

### Western blot analysis

A 15% non-denaturing PAGE gel was used to analyze the molecular weight of TDP species recognized by the FTD-TDP2 scFv. The resolving and stacking gels were prepared without SDS. 5X-Running buffer (15 g Tris + 72 g Glycine in 1L) and 2X-loading buffer (62.5 mM Tris–HCl, pH 6.8, 25% glycerol, 1% Bromophenol Blue) were also prepared without SDS detergent.

Protein samples were diluted in loading buffer and this mixture was loaded directly onto the gels without heat denaturation. Samples including two healthy control tissue samples, TDP-43 immunoprecipitated from two healthy controls and three different FTD individuals were analyzed. The gel apparatus was set at 70 V for 30 min followed by 100 V for approximately 3 h until the marker was well separated. A nitrocellulose membrane was used to transfer the separated bands from the gel using standard western protocol [58]. The blot was incubated at RT with 2% milk powder in 1X PBS followed by incubation with FTD-TDP2 scFv supernatant overnight at 4 °C. The blot was then washed with 1X PBS thrice followed by incubation with anti-c-myc (9e10) primary antibody (1:2000 dilution) for 2 h at RT. The blot was further washed with 1X PBS followed by incubation with goat anti-mouse HRP (1:1000 dilution) at RT for 45 min. After a final wash with 1X PBS, a colorimetric DAB substrate was added, and the blot was developed as per manufacturer’s protocol.

### Competition ELISA

To determine if the five FTD-TDP scFvs were binding to similar or different epitopes, a competition ELISA was performed as described [56, 59]. Each of the five FTD-TDP scFvs were pre-incubated with FTD sera at 37 °C for 1 h. During the addition of antigen, 1:100 dilution of FTD sera or FTD sera pre-incubated with FTD-TDP scFvs were used.

### Immunohistochemistry

Human postmortem tissue sections from superior frontal cortex were incubated with FTD-TDP2 and FTD-TDP3 scFvs respectively (1:100) on a shaking stage overnight at 4 °C. Primary antibodies against c-myc region of scFv (Sigma, 1:1000, rabbit) and MAP2 (Covance, 1:400, mouse) were applied to the tissue sections for 3 h at room temperature. Goat anti-rabbit IgG (green) and goat anti-mouse IgG (red) with fluorescence at the concentration of 1:1000 was used respectively as secondary antibodies for 1 h at room temperature. The sections were washed with PBS 3 times and the non-specific background was blocked with 0.03% Sudan black for 5 min. The sections were observed and imaged with Leica SP5. Commercial MAP2 antibody is visualized in red, anti-TDP scFv in green and DAPI, which stains the nucleus, in blue.

### Toxicity assay

TDP-43 for the toxicity assay was immunoprecipitated from human postmortem FTD and control brain tissue using four commercial antibodies–A16583 (cell signaling), ab190963 (Abcam), 10782-2-AP and 12892-1-AP (ProteinTech). The human neuroblastoma cell line, SH-SY5Y was used for toxicity studies. Cells were grown in serum free media on 6 well plates and once they reached confluence, toxicity was induced by incubating the cells with 1 µg/mL of TDP-43 IP from FTD or control. The cells were then incubated with commercial anti-TDP antibody ab190963 (Abcam, 1 µg/mL), or one of the anti-TDP scFvs–FTD-TDP1, FTD-TDP2, FTD-TDP3, FTD-TDP4 and FTD-TDP5. After 12 h of incubation, toxicity was measured using a lactate dehydrogenase assay kit [60].

### Statistical analysis

Luminescence signals obtained on the ELISAs were plotted as a ratio with respect to either background or healthy controls. Reactivity of each test sample was obtained relative to the average signal of the control group. Any sample with a ratio greater than 1 was considered a positive signal. Statistical significance was assessed using SPSS software (version 24) and one-way ANOVA with post hoc analyses was performed with p < 0.05. To determine the accuracy of the anti-TDP scFvs in detecting FTD over healthy controls, Receiver Operating Characteristic curves (ROC) and Area Under the Curve (AUC) were computed based on the reactivity of the five FTD-TDP scFvs with FTD-TDP, FTD-Tau and healthy control sera. Sensitivity and specificity of the FTD-TDP scFvs were also obtained by setting the cutoff as the average value of the healthy controls. 0.8 value for AUC is considered good while 0.5 (straight line) means it does not differentiate between FTD and control and is not a good diagnostic test.

## Supplementary information

**Additional file 1: Figure S1.** Western blot under denaturing conditions. Reactivity against healthy control tissue and TDP-43 immunoprecipitated from healthy controls and FTD was assessed under reducing and denaturing conditions with A) Commercial TDP antibody, and B) FTD-TDP2 scFv. While commercial antibody recognizes TDP variants in FTD and healthy controls, FTD-TDP2 scFv does not recognize TDP variants in any of the samples. **Figure S2.** Western blot under native conditions. Reactivity against healthy control tissue and TDP-43 immunoprecipitated from healthy controls and FTD was assessed under non-reducing conditions with A) Commercial TDP antibody, and B) FTD-TDP2 scFv. While commercial antibody recognizes TDP variants in FTD and healthy controls, FTD-TDP2 scFv does not recognize TDP variants in any of the samples.

## Data Availability

The dataset supporting the conclusions of this article is included within the article and additional file.

## Abbreviations

FTD: Frontotemporal dementia
TDP-43: TAR DNA Binding Protein 43
ALS: Amyotropic lateral sclerosis
scFv: Single chain variable fragment
Aβ: Amyloid beta
AFM: Atomic force microscopy
BSA: Bovine serum album
BCA: Bicinchoninic acid
IPTG: Isopropyl β-d-1-thiogalactopyranoside
IgG: Immunoglobulin G
ELISA: Enzyme linked immunosorbent assay
EDTA: Ethylenediaminetetraacetic acid
SDS-PAGE: Sodium dodecyl sulfate–Poly Acrylamide Gel Electrophoresis
DAPI: 4′,6-diamidino-2-phenylindole
One-way ANOVA: One-way analysis of variance
