# Supplementary material for: Isolation and characterization of antibody fragments selective for human FTD brain derived TDP-43 variants
Source: BMC Neurosci. 2020 Sep 4;21:36. doi: 10.1186/s12868-020-00586-0 (PMC7472585; doi:10.1186/s12868-020-00586-0)
Supplement: Supplementary file 1 — Additional file 1: Figure S1. Western blot under denaturing conditions. Reactivity against healthy control tissue and TDP-43 immunoprecipitated from healthy controls and FTD was assessed under reducing and denaturing conditions with A) Commercial TDP antibody, and B) FTD-TDP2 scFv. While commercial antibody recognizes TDP variants in FTD and healthy controls, FTD-TDP2 scFv does not recognize TDP variants in any of the samples. Figure S2. Western blot under native conditions. Reactivity against healthy control tissue and TDP-43 immunoprecipitated from healthy controls and FTD was assessed under non-reducing conditions with A) Commercial TDP antibody, and B) FTD-TDP2 scFv. While commercial antibody recognizes TDP variants in FTD and healthy controls, FTD-TDP2 scFv does not recognize TDP variants in any of the samples. [file 12868_2020_586_MOESM1_ESM.pptx]

## Slide 1
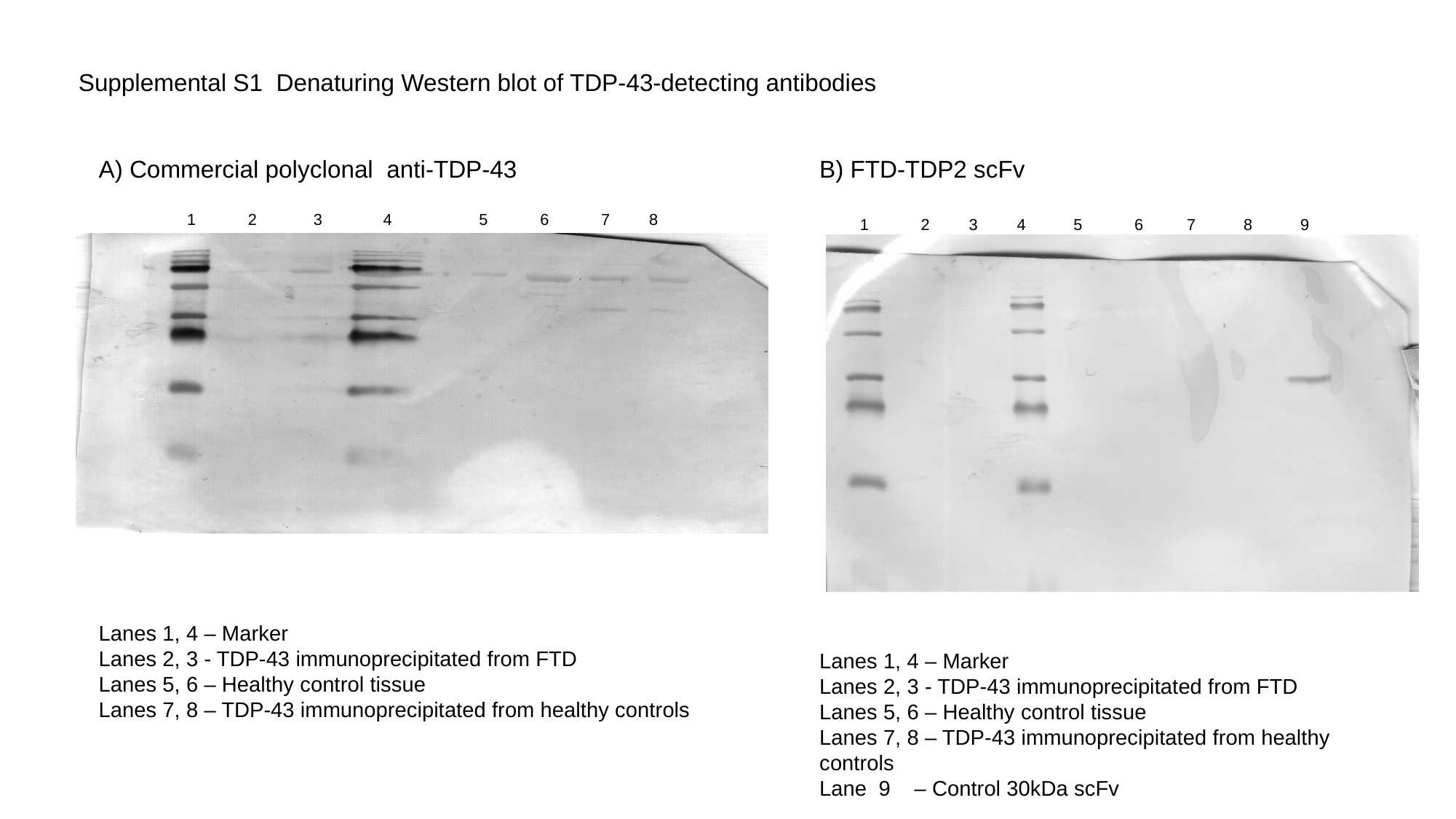

Supplemental S1 Denaturing Western blot of TDP-43-detecting antibodies
B) FTD-TDP2 scFv
A) Commercial polyclonal anti-TDP-43
1 2 3 4 5 6 7 8
1 2 3 4 5 6 7 8 9
Lanes 1, 4 – Marker
Lanes 2, 3 - TDP-43 immunoprecipitated from FTD
Lanes 5, 6 – Healthy control tissue
Lanes 7, 8 – TDP-43 immunoprecipitated from healthy controls
Lanes 1, 4 – Marker
Lanes 2, 3 - TDP-43 immunoprecipitated from FTD
Lanes 5, 6 – Healthy control tissue
Lanes 7, 8 – TDP-43 immunoprecipitated from healthy controls
Lane 9 – Control 30kDa scFv

## Slide 2
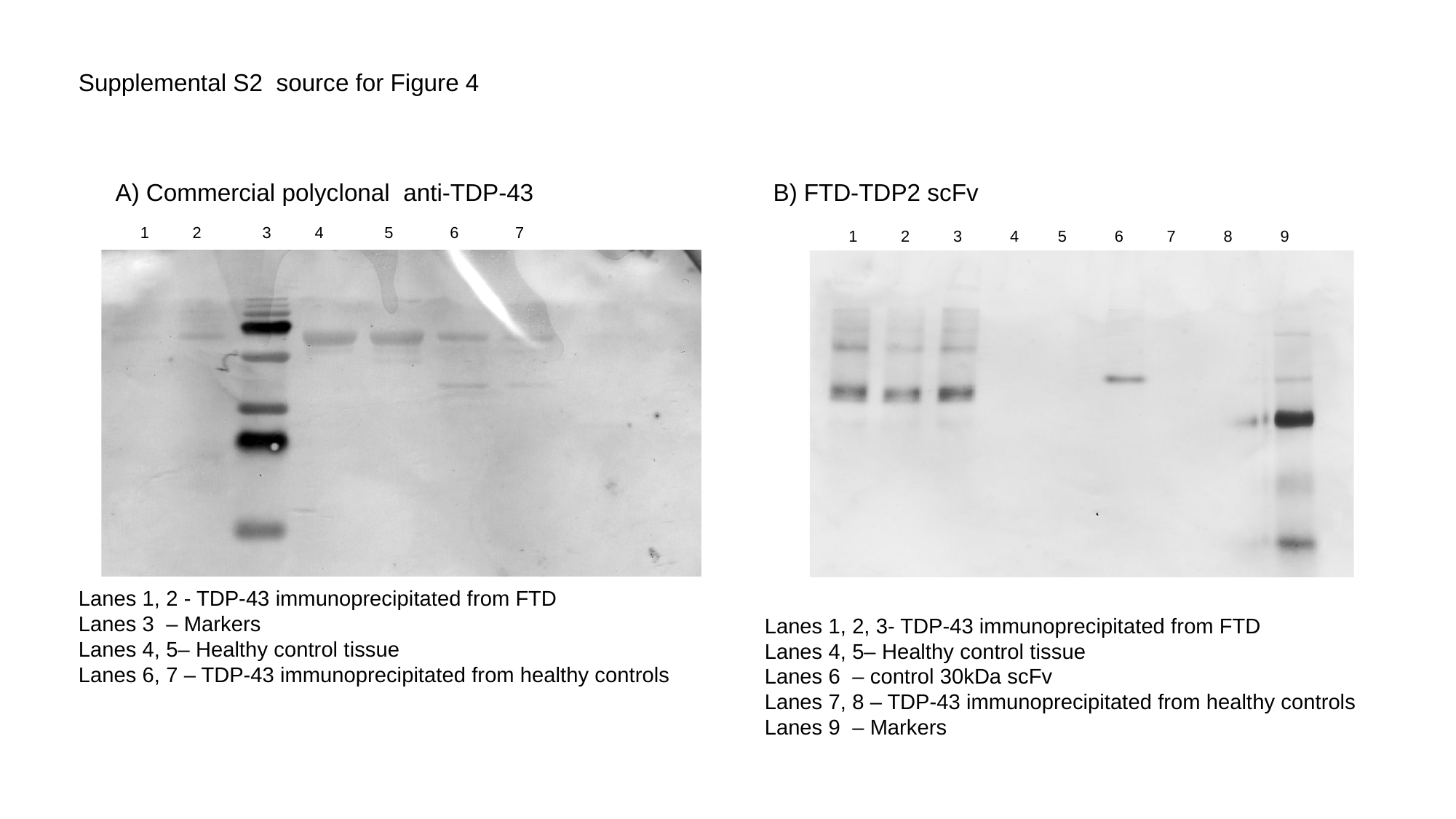

Supplemental S2 source for Figure 4
B) FTD-TDP2 scFv
A) Commercial polyclonal anti-TDP-43
1 2 3 4 5 6 7
1 2 3 4 5 6 7 8 9
Lanes 1, 2 - TDP-43 immunoprecipitated from FTD
Lanes 3 – Markers
Lanes 4, 5– Healthy control tissue
Lanes 6, 7 – TDP-43 immunoprecipitated from healthy controls
Lanes 1, 2, 3- TDP-43 immunoprecipitated from FTD
Lanes 4, 5– Healthy control tissue
Lanes 6 – control 30kDa scFv
Lanes 7, 8 – TDP-43 immunoprecipitated from healthy controls
Lanes 9 – Markers
